# Annotations

**Published:** 1890-04-19

**Authors:** 


### Annotations.

**Epsom College.** The Biennial Festival Dinner of Epsom College, which took place on Thursday at the Hotel Metropole, under the presidency of Sir James Paget, F.R.S., brings afresh to recollection an institution of much value to medical men *in actu* as well as to those *in posse*. Epsom, to the medical mind, is not associated with the race-course, but with sundry quiet and pleasant homes to which aged medical men may retire who have been somewhat over-matched in their struggle with the world; and still more with the "College," to which so many embryo and fully-fledged doctors look as their best-beloved *alma mater*. Epsom is, and is not, a charitable institution. It is charitable in that it provides homes and pensions for aged medical men and their widows; and in that it receives into its "halls of learning," as "foundationers," the sons of medical men who have had the misfortune to be orphaned in early life. Such an institution has its parallels at Haileybury and Wellington. Those three, and other similar schools, render to the "professions" which they represent services invaluable. The death-bed of many a doctor who has passed away long before fame and fortune came knocking at his door has been made more tolerable by the thought that Epsom would receive his boys, would guard them with almost paternal care, would train them for the position their father would have wished them to fill, and would give them unfailing counsel and help until help should no longer be required. Epsom is a truly brotherly institution; it is every doctor's friend. Not only does it give aid to those who are in distress, but it offers one of the readiest and happiest media by which the prosperous may help the unprosperous without patronage on the one side or loss of self-respect on the other. The college, we have every reason to believe, is a very flourishing institution, though we cannot doubt that if the public and the wealthier members of the profession were to furnish more liberal resources the managers would gladly enlarge their borders and enter upon new fields of usefulness. It is saying little to affirm that Epsom occupies a unique place in both the confidence and the affection of the whole medical profession. Let us hope that material aid corresponding to this affection and confidence will be liberally poured into her treasury.

**A Gleam of Sense.** At the recent conference of the Teachers' National Union, the following resolution was agreed to: "That the annual examination in elementary schools ought to be abolished, and that all schools in receipt of Government grant should be open at any time to her Majesty's inspectors." There is thus at least a promise of the dawn of a brighter day for young children. The Education Department has, with the best intentions, produced a vast amount of suffering and misery among the children of every town and village in the kingdom, and at the same time has succeeded in injuring almost as many youthful brains as it has helped. The Department has acted with zeal and energy but not with wisdom. Public authorities are conscious that they are brought into existence to do something, and they do it. They have no faith whatever in the old-fashioned principle that it is better to do nothing at all than to do that which is worse than nothing. The periodical examinations of young children, with the payment of teachers by results, have led to methods of preparation—we will not call it "education"—that have been very much worse than nothing. It is one of the foolishest things imaginable to cram boys and girls under ten or twelve years of age for formal examination. The injury done to the children is very great; that done to the teachers is almost greater still. The former are so worried by "cram" that they cannot learn; the latter are so harassed by anxiety about results that they cannot teach. Let every practical politician ask himself what it is that elementary schools ought to do for the masses of the population? They should teach children how to speak; for correct speaking enables a person to communicate easily with all classes of his fellow-men. They should teach them how to write; for writing enables a person to preserve the ties of friendship and of relationship in every quarter of the world. They should teach them the elements of arithmetic, because various kinds of money have taken the place of goods in bargains between man and man; and the grown up man cannot do properly that which civilization demands of him without a knowledge of elementary arithmetic. They should teach them how to read; for reading enables a man to understand what the world has been both doing and thinking in past times, and also to know what the best minds in the world regard as the true and proper duty of human beings. Above all they should teach them how to think honestly, strongly, and courageously for themselves. Everybody can see that elementary education conducted on these lines will make serviceable and intelligent men and women, exactly as the proper breaking of a horse trains him to carry weight, to draw a carriage, to plough, and to do everything else that is useful. The Education Department, it seems to us, has been aiming at making circus horses instead of horses for the plough, the cart, and the saddle. It is not the business of the Department to do any such thing. Parents don't wish it, children don't wish it, teachers don't wish it, and the public does not wish it. Let us have plain elementary schools for the masses; and let them be taught in such a way as that both brain and body may be developed and strengthened, and that harassing care may be avoided until the age arrives when care can be properly faced and endured. Secondary schools let us have by all means for the cleverest boys and girls, even of the lowest ranks. But for those who must always be "hewers of wood and drawers of water," let us have such a training as will enable them both to hew wood and to draw water with the greatest advantage alike to themselves and their employers. It is quite impossible "to make a silk purse out of a sow's ear"; and the man or the department that spends money and energy in persistent attempts at it, merely shows how excessively stupid even "educated" and "public" men can be when they try.

**Popular Errors.** The general public have not infrequently a most elementary knowledge of matters closely affecting their interests. The influenza epidemic has especially emphasized this fact in one direction—that of nursing. An opinion seems to generally prevail that no nurses are obtainable from the principal hospitals, but that they have to be sought elsewhere if a good nurse is to be secured. Yet the principal hospitals with nurse training schools now have nursing institutions attached to them from which excellent nurses may be procured by the public on application to the matron. In the same way the arguments in favour of the registration of nurses are based upon the popular error that nothing can prevent any woman proclaiming herself a trained nurse under existing circumstances. It is further erroneously believed that no means of withdrawing a certificate, however much a nurse may disgrace herself by misconduct, exists at the present time. So far from this being the case, any member of the public who employs a nurse can demand the production of her certificate of training, and should she misconduct herself, can address a letter to the matron of the institution where she was trained, which will result in a thorough investigation and the enforcement of adequate punishment. Despite these facts advocates of nurse-registration have had the effrontery to declare that even if a woman suffers imprisonment she could, on regaining her liberty, victimize the public by trading on her certificate, whereas communication to the authorities of the school where she was trained would effectually prevent any such misconduct. Although nursing is not a profession, but a calling, being, in fact, the handmaiden of medicine, it is desirable that competent nurses and the public should be protected against the incompetent. Adequate protection against all abuses is provided at the present time by the issue of certificates under the sign-manual of the nurse training schools throughout the country, which certificates can always be produced to employers, who should make a note of the date and the school where each nurse was trained. The British Nurses' Association cannot issue certificates of training, or give the public any guarantee whatever of the moral or individual efficiency of a nurse, whilst admission to the voluntary register, opened under the auspices of this association must result in the whitewashing of a number of really inefficient and uncertificated nurses. A knowledge of this last fact has made every prudent nurse who has a certificate decline to permit her name to appear on the Register of the British Nurses' Association.

